# Lateral versus posterior surgical approach for the treatment of supracondylar humeral fractures in children: a systematic review and meta-analysis

**DOI:** 10.12688/f1000research.53599.2

**Published:** 2021-11-18

**Authors:** Komang Agung Irianto, I Putu Gede Pradnyadewa Pradana, Brigita De Vega

**Affiliations:** 1 Department of Orthopaedics and Traumatology, Airlangga University, Surabaya, Indonesia, 60286, Indonesia; 2 Institute of Orthopaedics & Musculoskeletal Science Division of Surgery & Interventional Science, University College London, Brockley Hill, Standmore, HA7 4LP, UK

**Keywords:** supracondylar fractures, humeral fractures, lateral approach, posterior approach, children

## Abstract

**Background:** Supracondylar humeral fracture (SHF) is the most common type of fracture in children. Moreover, lateral and posterior surgical approaches are the most frequently chosen approaches for open reduction surgery in displaced SHF when C-arm is unavailable. However, previous literature showed mixed findings regarding functional and cosmetic outcomes. Currently, no systematic review and meta-analysis has compared these two procedures.

**Methods:** Our protocol was registered at PROSPERO (registration number CRD42021213763). We conducted a comprehensive electronic database search in MEDLINE, EMBASE, and CENTRAL. Two independent reviewers screened the title and abstract, followed by full-text reading and study selection based on eligibility criteria. The quality of the selected studies was analyzed with the ROBINS-I tool. Meta-analysis was carried out to compare the range of motion (functional outcome) and cosmetic outcome according to Flynn’s criteria. This systematic review was conducted based on PRISMA and Cochrane handbook guidelines.

**Results:** Our initial search yielded 163 studies, from which we included five comparative studies comprising 231 children in the qualitative and quantitative analysis. The lateral approach was more likely to result in excellent (OR 1.69, 95% CI [0.97-2.93]) and good (OR 1.12, 95% CI [0.61-2.04]) functional outcomes and less likely to result in fair (OR 0.84, 95% CI [0.34-2.13]) and poor (OR 0.42, 95% CI [0.1-1.73]) functional outcomes compared to the posterior approach. In terms of cosmetic results, both approaches showed mixed findings. The lateral approach was more likely to result in excellent (OR 1.11, 95% CI [0.61-2.02]) and fair (OR 1.18, 95% CI [0.49-2.80]) but less likely to result in good (OR 0.79, 95% CI [0.40-1.55]) cosmetic outcomes. However, none of these analyses were statistically significant (p> 0.05).

**Conclusion:** Lateral and posterior surgical approaches resulted in satisfactory functional and cosmetic outcomes. The two approaches are comparable for treating SHF in children when evaluated with Flynn’s criteria.

## Introduction

Supracondylar humeral fracture (SHF) is an elbow injury in children that most often requires surgical therapy. Nearly 60-70% of all elbow injuries occur in children aged five to seven. ^1^ In the USA, it is reported that 25-40% of the incidence of SHF occurs in children's playgrounds. ^2^ From all the injuries to the elbow joint that can occur in children, 85% occurs in the distal humerus area, and 55-75% of the total fractures of the distal humerus are fractures of the supracondylar humerus. The annual incidence of SHF in the US is estimated at 177.3 per 100,000 children. Moreover, the incidence of trauma as the leading cause of SHF has a seasonal distribution. The literature shows that SHF is more frequent in the summer and is more common in the left elbow or non-dominant limb. ^3^ In addition, SHF may give rise to an emergency, namely compartment syndrome. This life and limb-threatening complication occurs in 0.3-1% of all SHF cases. A study conducted by Houshian et al. shows that elbow fracture incident is 308/100,000 per year; supracondylar humeral fracture comprises 58% of all incidents. ^4^^,^^5^

Treatment choices for SHF in children can be classified into non-operative and operative treatment. In cases where the fracture configuration is not displaced (Gartland type 1) or minimally displaced (Gartland type 2), the primary treatment choice is non-operative. Whereas, in displaced fractures (Gartland type 3 or 4), the gold standard is operative treatment with closed reduction techniques and percutaneous pining with Kirschner wire insertion (CRPP). However, when these attempts fail, the next step is to perform an open reduction and internal fixation (ORIF) to obtain an optimal reduction. Anatomical restoration of displaced fractures is important since it will affect the outcome on the function of the elbow joint. ^6^^,^^7^ Nevertheless, CRPP is often unfeasible in regions/countries with limited settings due to the unavailability of intraoperative fluoroscopic imaging (i.e., C-arm). Thus, open reduction surgery is the only choice in this situation. ^31^

There are various surgical approaches in open reduction surgery for SHF. Several approaches that are commonly used are lateral, medial, anterior and posterior approaches. As they can only provide visualization to some part of the fracture site, a combination of them is often performed. The three operating approaches (lateral, medial, posterior) can include fairly straightforward operating technique, except the anterior approach. A lateral approach is often used because this approach causes relatively less destruction to important neurovascular structures and soft tissues but still gives enough exposure to reposition the fracture site. Sometimes, this approach can be combined with a medical approach if necessary, but that will add another scar and also extensive soft tissue exposure. Another approach that can be performed is the anterior approach, which possesses several advantages, one of which is evacuating the hematoma at the fracture site because usually, the hematoma will accumulate in the anterior side of the fracture site. However, the downside of the anterior approach is its challenging technique because there will be quite a number of neurovascular structures that require extra caution, making the surgery less straighforward. ^8^ Moreover, the posterior surgical approach is also often used due to its simplicity as it does not require much neurovascular structure identification, resulting in a shorter surgery time. Thus, the anterior approach is technically more demanding than the posterior approach, especially because simultaneous reduction and manipulation of the K-wires seems challenging. ^31^

To date, there is no definite consensus on which operating approach should be used as the main approach in open repositioning surgery for SHF treatment in children. Thus, it is our aim to carry out a comprehensive systematic review to determine the best surgical approach in SHF, especially comparing those that are often chosen practiced worldwide (including in limited settings), namely the posterior and lateral surgical approaches, in order to achieve the goal of high patient satisfaction.

## Methods

### Protocol and registration

We followed PRISMA and Cochrane handbook guidelines for conducting a systematic review of interventions. Our protocol has been registered at PROSPERO (registration number CRD42021213763).

### Eligibility criteria

This research is a systematic review and meta-analysis that includes a direct comparative study between lateral and posterior surgical approaches for supracondylar humeral fractures (SHF) in children. We included original clinical studies which were written in English and available in full-text. However, we did not include the following types of articles: abstract conferences, letters to editors, summaries of meetings, expert opinions, book chapters, study protocols, technical reports, narrative reviews, systematic reviews, meta-analyses, case reports, studies with incomplete data, duplication of publications, experimental studies on animals, and cadavers, laboratory ( *in vitro*), and computational studies.

The population used in this study were children diagnosed with supracondylar humeral fractures who underwent open reduction and internal fixation surgery using a lateral or posterior surgical approach. Patients who underwent conservative treatment or surgeries with other approaches were not included in this review. We also excluded the studies that used combined approaches. The primary outcome assessed in this study was functional and cosmetic outcomes according to Flynn's criteria ^9^ ( Table 1).

**Table 1.** Flynn’s criteria. ^9^

Result	Rating	Functional factor: Motion loss (Degrees)	Cosmetic factor: Carrying-angle loss (Degrees)
Satisfactory	Excellent Good Fair	0-5 5-10 10-15	0-5 5-10 10-15
Unsatisfactory	Poor	Over 15	Over 15

### Literature search

We conducted an electronic literature search in MEDLINE (from 1966 until 1 November 2020), EMBASE (from 1947 until 1 November 2020), and The Cochrane Central Register of Controlled Trials (CENTRAL) (from inception until 1 November 2020) using free-text keywords and subject subheading (Medical Subject Headings (MeSH) for MEDLINE and Emtree for EMBASE). Our search strategy can be seen in Extended Data. ^30^ The PICO concept (Patient, Intervention, Comparison, Outcome) was used in conceptualizing this research strategy:

• P: children diagnosed with supracondylar humeral fractures requiring open reduction and internal fixation surgeries,
• I: open reduction and internal fixation with a lateral or posterior approach,
• C: as explained in Intervention (I),
• O: Fylnn’s criteria evaluation.

### Selection process and data extraction

Study references obtained from the search were transferred to Mendeley software version 1.19.8 (Elsevier, United Kingdom) to detect duplicates. Two independent reviewers screened the references by title and abstract based on the eligibility criteria using Rayyan ^®^ webpage, ^10^ and labelling was carried out using ‘include’, ‘maybe’, and ‘excluded features’. Potentially eligible studies or studies that remained unclear were included in the full-text reading. Any disagreement that arose was resolved by a third reviewer. Studies that were excluded at the full-text reading stage were recorded and provided with reasons. The flow of the study selection process is presented in the PRISMA flow diagram.

The selected studies were extracted using Microsoft Excel ^®^ software. We collected the following data: author, year of publication, title, type of study, patient demographics (sex, age), SHF classification, follow-up duration, surgical approach, functional and cosmetic outcome based on Flynn's criteria, and authors’ conclusions.

### Bias analysis

Bias analysis was carried out using the risk of bias tools formulated by the Cochrane group. For Randomized Controlled Trial (RCT) studies, we used the second version of the Cochrane tool, Risk of Bias (ROB). ^11^ Potential causes of bias were assessed with signaling questions to detect biases caused by the randomization process, deviation from initial intervention intent, missing data, measurement of outcomes, and reporting of selective bias. For non-RCT studies, we used ROBINS-I (Risk of Bias in Non-randomized Studies of Intervention). ^12^ The component of the assessment was the same as the measurement of bias using the ROB, but there were additional biases such as bias in patient selection and bias due to confounding factors. Meta-analyses were conducted only with studies that had a moderate or better risk of bias.

### Synthesis of results

We assessed odds ratios (ORs) with a 95% confidence interval (CI) for the data. Heterogeneity (inconsistency) was analyzed using the Chi ^2^ and I ^2^ tests. A low p-value result (p < 0.1) of the Chi ^2^ test indicates significant heterogeneity. Because the Chi ^2^ test has a low detection ability in a small sample of data, we also used the I ^2^ test to assess heterogeneity. An I ^2^ test score of more than 50% has significant heterogeneity. Statistical analyses were performed using the Review Manager (RevMan) ^®^ version 5.3 (Nordic Cochrane Center, Denmark). If the heterogeneity test results showed no significant heterogeneity, we planned to use the fixed-effect models. Otherwise, the researchers used random-effect models to process the data. Subgroup analyses were also planned to explore the causes of high heterogeneity ^13^ based on the type of study (RCT and non-RCT). When we encountered an unclear (inconclusive) decision, we carried out a sensitivity analysis test by repeating the meta-analysis with other effect magnitudes (risk ratio/RR, odds ratio/OR, and mean difference) and alternative statistical models (fixed and random effects models). ^12^ To ensure a reliable meta-analysis result, we did not include studies that have a high risk of bias.

## Results

### Selection process

**Figure 1.**
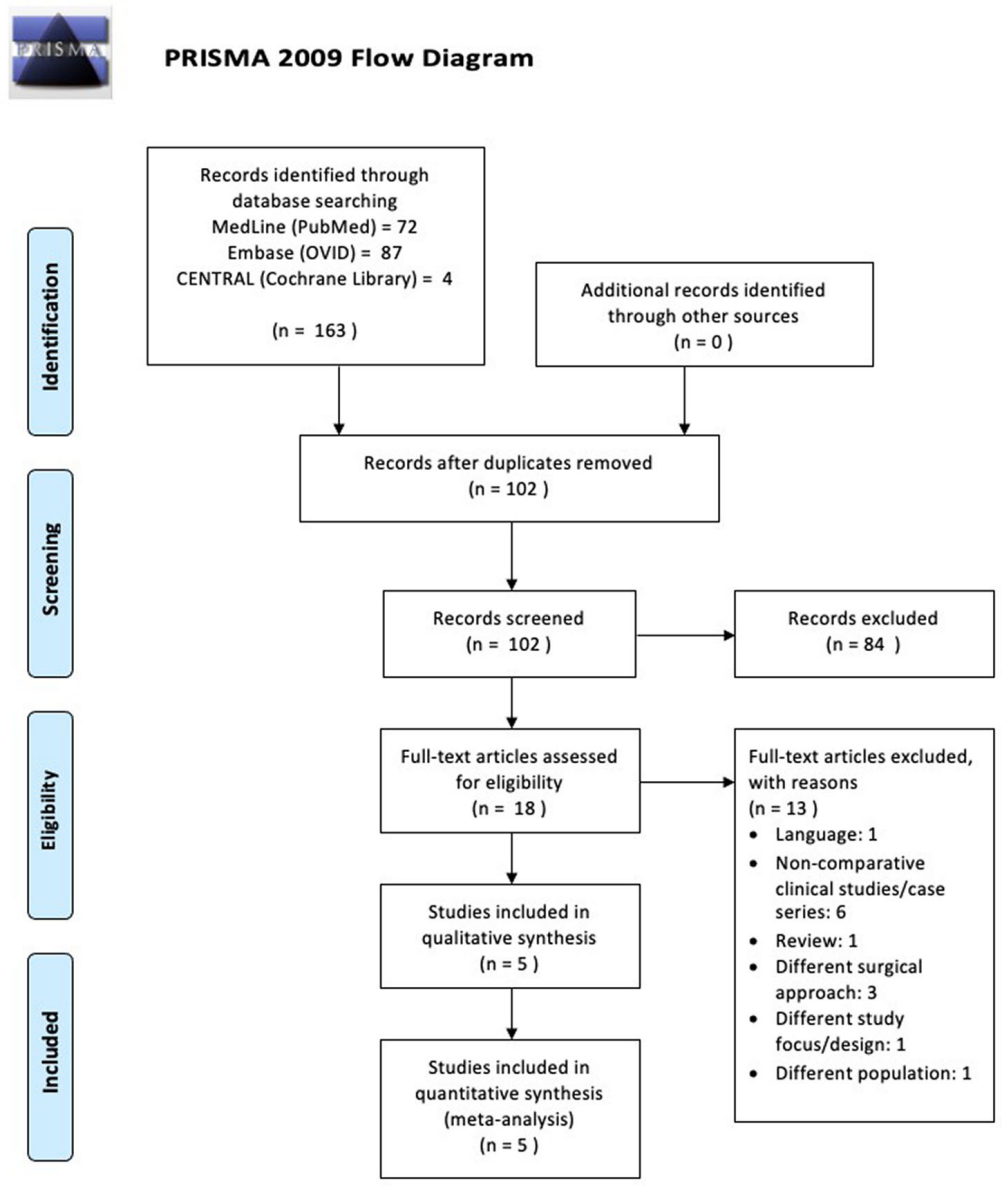
The workflow of this review.

Our initial electronic search results yielded 163 studies that matched the search keyword algorithm in the three major databases. The duplication removal process resulted in a total of 102 studies. The remaining studies were then screened by title and abstracts that had conformity to the inclusion and exclusion criteria. At this stage, we excluded 84 studies that were deemed irrelevant. The remaining 18 studies were then read as full-text articles to assess their suitability for this study. We excluded 13 studies due to several reasons: six studies were case series studies; three studies used a different operating approach from the main study; one study was an article review; one study was found to be not in accordance with the objectives of this study; one study was not supracondylar humeral fractures (different population); and one last study was not in English. Finally, five studies that were suitable for this review were chosen. A brief description of the study search is presented in Figure 1.

### Description of the included studies

The five studies included were all retrospective, case-control studies conducted in Turkey, ^14^^–^^16^ Thailand, ^17^ and France ^18^ between 2004 and 2013. The earliest study was initiated by Bamrungthin in 2004, ^17^ while the latest study was conducted by Basaran et al *.* ^18^ and Uzer et al *.* ^14^ in 2010. The total number of patients in this study was 231 patients. One study ^15^ did not specify the sex distribution of the patients. Of the five other studies included in this study, it was found that 119 patients (62%) were male, and 72 patients (38%) were female. All patients were children diagnosed with displaced supracondylar humeral fractures.

All patients in these studies had surgical management performed in the operating room using either a lateral or posterior operative approach to perform open repositioning of the fracture and then fixed with Kirschner wire to maintain the repositioning. After being operated on, the operated limb was supported with a back slab. All patients in this study were subjected to periodic examinations at the polyclinic for functional and cosmetic measurements using Flynn's criteria. The follow-up time in each study was different, ranging from 6-7 months ^18^ until up to 50 months ^16^ following the surgery.

### Risk of bias analysis

Overall, the five included studies had a moderate risk of bias. The summary of the risk of bias analysis using ROBINS-I tool can be seen in Table 2.

**Table 2.** Risk of bias of the included studies.

Author, Year	Bias due to confounding	Bias in selection of participants into the study	Bias in classification of intervention	Bias due to deviation from intended interventions	Bias due to missing data	Bias in measurement of outcomes	Bias in selection of the reported result	Overall risk of bias
Bamrungthin, 2008	Low	Low	Low	No information	Low	Low	Moderate	Moderate
Basaran et al., 2014	Low	Moderate	Low	No information	Low	Low	Moderate	Moderate
Kizilay et al *.,* 2017	Low	Moderate	Low	No information	Low	Low	Moderate	Moderate
Turkmen et al., 2016	Low	Moderate	Low	No information	Moderate	Low	Moderate	Moderate
Uzer et al., 2017	Low	Low	Low	No information	Low	Low	Moderate	Moderate

• Confounding bias

This bias can arise if the interventions and outcomes have a different relationship from the cause due to confounding factors. Examples of common confounding factors are the presence of comorbid diseases and differences in socioeconomic status (including access to health insurance), which affect changes in the choice of intervention and changes in outcomes that are different from conditions in general. ^12^^,^^13^ In this study, the authors found there was no risk of confounding factors.

• Bias in the selection of research subjects

The author found there were two studies ^14^^,^^17^ that had a low risk of bias in the selection of study subjects. In these studies, the researchers included all patients who met the inclusion criteria (patients with displaced supracondylar humeral fractures who were operated on with open reduction and percutaneous K-wire fixation posteriorly and laterally). Meanwhile, the other three studies ^15^^,^^16^^,^^18^ had moderate bias because, despite the well-described inclusion criteria and subject selection flow, the number and reasons for exclusion of some subjects were not well defined. ^12^^,^^13^ Therefore, the authors judged the possibility of bias in the selection of research subjects in these four studies.

• Bias in the classification of the intervention group

Bias in the classification of the intervention group is low if the definition of the intervention is well explained and the definition of the intervention is only based on the information gathered at the time of the intervention (not determined later). ^12^^,^^13^ The authors assigned a low degree of bias to this category because all studies adequately explained the definition and operational approach of the two intervention groups. Additionally, the definition of the intervention was taken at the time the operation was performed (recorded in the operation log), thereby reducing the risk of bias. Therefore, the authors found no bias in misclassifying the intervention group.

• Bias due to deviations from previously planned interventions

Deviations from previously planned interventions can occur when the patient does not adhere to the prescribed intervention or when the patient can change/switch intervention groups during the study period (in other words, there is a protocol violation). ^12^^,^^13^ The authors considered this bias as not having sufficient information because all studies included in this study were retrospective studies of medical records, and there was no predetermined protocol. Therefore, it was quite difficult to judge whether there was a protocol violation or not.

• Bias due to missing/incomplete data

This type of bias occurs when a large number of patients experiences loss of follow-up after they are included in the study (90-95% data availability is deemed sufficient), or when participants are excluded from the analysis by the principal investigator without clear justification/reasons. ^12^^,^^13^ The study conducted by Turkmen et al *.* ^16^ was considered to have a moderate bias due to incomplete data. Investigators in this study noted that they excluded a number of patients due to incomplete data or loss to follow-up but did not adequately explain the numbers. The other four studies ^15^^,^^16^^,^^18^^,^^19^ were considered to have a low bias because all the required data were presented in full.

• Bias in outcome measures

Bias in the measurement of results is categorized as low if it meets the following criteria: (i) the method of measuring the results used by all groups is equal and equivalent; (ii) the measurement of the results is objective (cannot be influenced by the knowledge of the type of intervention received by the research subject) or the outcome assessor does not know the type of intervention received by the research subject; and (iii) the existence of an error in measuring the results is not related to the intervention. ^12^^,^^13^ The authors considered the five studies as having low bias because they met all of the above criteria.

• Bias in the selection of reported results

The authors found that all studies included in this study had a moderate bias in the selection of reported results. This bias was assessed by matching the results of reports or scientific articles published in peer-reviewed journals with the protocols registered before the study was conducted (for example, registering clinical studies on clinicaltrial.gov). ^12^^,^^13^ Since all five studies are retrospective, there will always be a bias in this category. However, the risk of bias was categorized as moderate (not serious/high risk) because, in the methodology section, all studies clearly explained the method of measurement and statistical analysis used.

### Qualitative synthesis

The patient demographic and surgery outcomes of the included studies are presented in Tables 3 and 4.

**Table 3.** Patient demographic. L: Lateral surgical approach, P: Posterior surgical approach, NI: No information.

Author, Year of Publication	Country	Study period	Study design	Patient demographic							Fracture classification	Follow-up duration (Months)	
				Total patients	Male		Female		Age (years)				
					L	P	L	P	L	P		L	P
Bamrungthin, 2008	Thailand	2004-2007	Retrospective, Case-Control	82	18	34	12	18	7.2 ± 3.2	6.04 ± 1.5	Gartland Type 3	7.0 ± 1.2	7.6 ± 2.2
Basaran et al *.,* 2014	France	2010-2011	Retrospective, Case-Control	24	11	8	1	4	3.7 ± 1.7	7 ± 2.8	Gartland Type 3	8.1 ± 2.6	7.5 ± 2.5
Kizilay et al *.,* 2017	Turkey	2007-2012	Retrospective, Case-Control	40	NI	NI	NI	NI	6.87 ± 2.81		Gartland Type 3	31.71 ± 16.5	31.71 ± 16.5
Turkmen et al *.,* 2016	Turkey	2008-2013	Retrospective, Case-Control	38	5	18	3	12	7.9 (range 5.1–12.7)	7.8 (range 5.1–12.7)	Gartland Type 2/3	50.32 (range 16.8–86.4)	50.41 (range 16.8–86.4)
Uzer et al., 2017	Turkey	2010-2012	Retrospective, Case-Control	47	14	11	8	14	7.3 (range 2–13)	7.3 (range 2.5–14)	Gartland Type 3	12	12

**Table 4.** Patient Outcomes based on Fylnn’s criteria. L: Lateral surgical approach, P: Posterior surgical approach.

Author, Year of Publication	Flynn's Criteria																Satisfactory result				Author’s conclusion
	Excellent				Good				Fair				Poor								
	Functional		Cosmetic		Functional		Cosmetic		Functional		Cosmetic		Functional		Cosmetic		Functional		Cosmetic		
	L	P	L	P	L	P	L	P	L	P	L	P	L	P	L	P	L	P	L	P	
Bamrungthin, 2008	17	32	18	34	7	10	6	10	5	8	5	7	1	2	1	1	29	50	30	51	No significant difference
Basaran et al., 2014	5	5	9	10	7	3	2	2	0	1	1	0	0	3	0	0	12	9	12	12	No significant difference
Kizilay et al., 2017	7	6	11	21	3	16	0	7	0	3	0	1	1	4	0	0	10	25	11	29	No significant difference
Turkmen et al *.,* 2016	6	21	6	21	2	9	2	9	0	0	0	0	0	0	0	0	8	30	8	30	No significant difference
Uzer et al *.,* 2017	13	17	12	13	7	6	6	7	2	2	4	5	0	0	0	0	22	25	22	25	No significant difference

**Figure 2.**
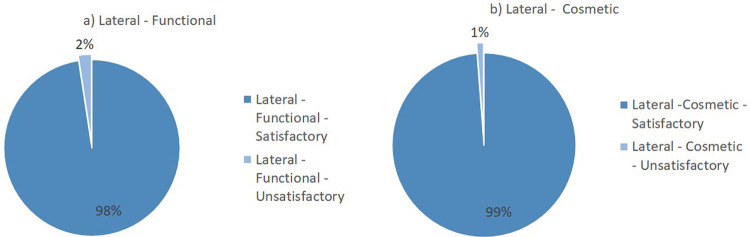
Outcomes of the lateral surgical approach based on Flynn’s criteria: (a) functional outcome and (b) cosmetic outcome.

**Figure 3.**
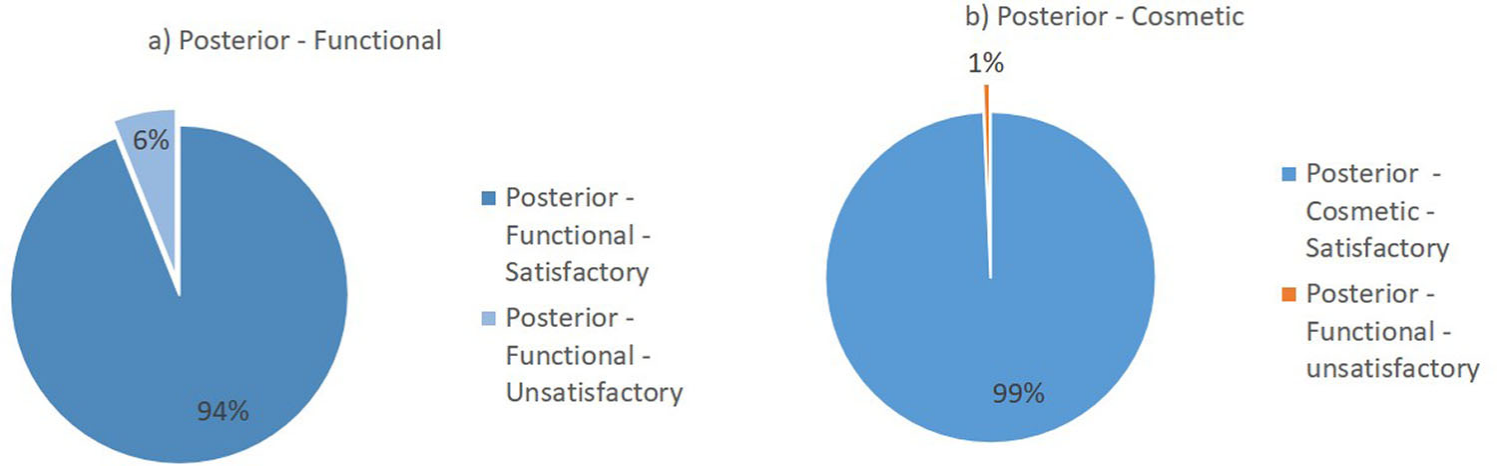
Outcomes of the posterior surgical approach based on Flynn’s criteria: (a) functional outcome and (b) cosmetic outcome.

Flynn’s criteria divides the results of the evaluation into two major groups: satisfactory and unsatisfactory. The summary of the lateral surgical approach outcomes from the included studies is presented in Figure 2. Overall, the lateral surgical approach resulted in 98% functional satisfaction and 99% cosmetic satisfaction. Meanwhile, the posterior surgical approach resulted in 94% functional satisfaction and 99% cosmetic satisfaction ( Figure 3).

### Quantitative synthesis

Meta-analyses were conducted to quantify the difference between the two surgical approaches. We divided the meta-analyses based on the subgroup grading of Flynn’s criteria (excellent, good, fair, poor). Overall, all of the data included in the meta-analyses had low heterogeneity (I ^2^ < 50%), which means that our data were consistent.

Functional outcome

**Figure 4.**
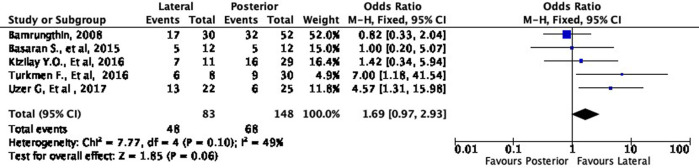
Comparison of functional outcome in the excellent subgroup.

**Figure 5.**
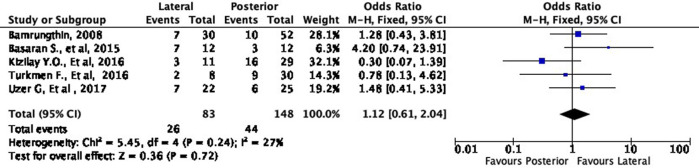
Comparison of functional outcome in the good subgroup.

Figure 4 shows the functional outcome of the meta-analysis in the excellent subgroup, while Figure 5 shows the meta-analysis’ functional outcome in the good subgroup. The lateral approach was 69% (OR 1.69, 95% CI [0.97-2.93]) and 12% (OR 1.12, 95% CI [0.61-2.04]) more likely to result in excellent and good results, respectively, compared to the posterior approach. However, these differences were not statistically significant (p = 0.06 and p = 0.72, respectively).

**Figure 6.**
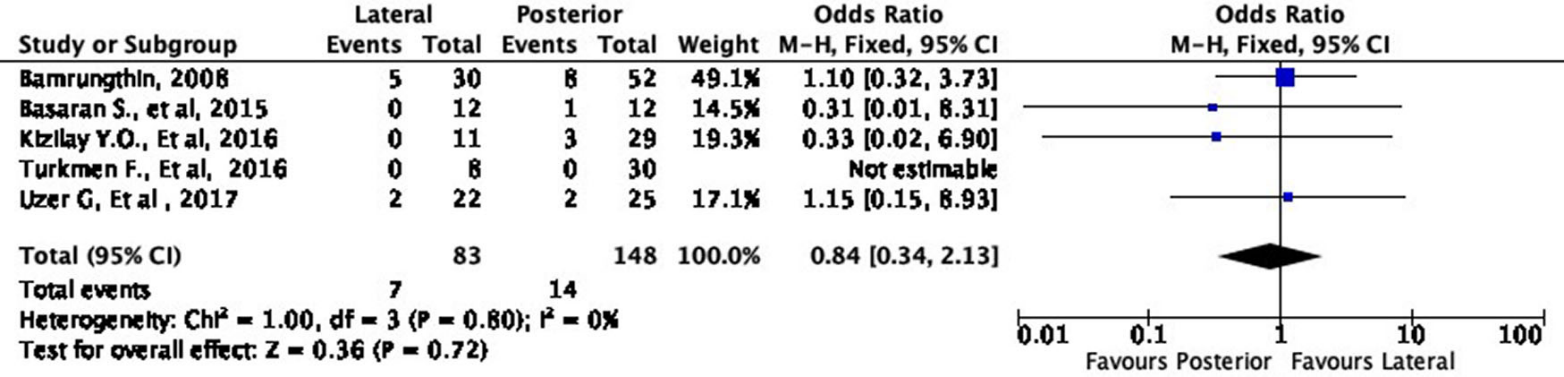
Comparison of functional outcome in the fair subgroup.

**Figure 7.**
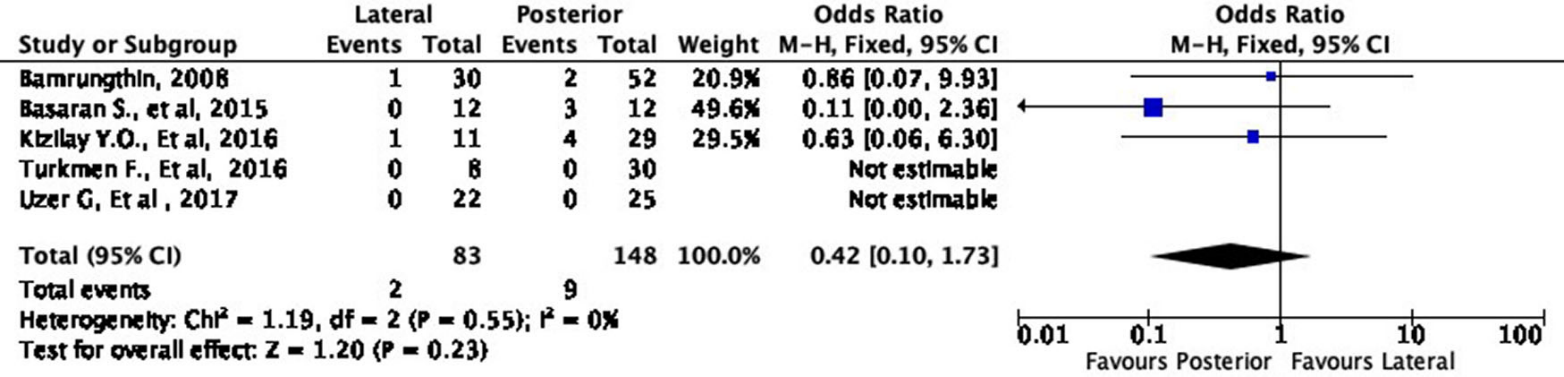
Comparison of functional outcome in the poor subgroup.

Moreover, the lateral approach resulted in lower fair and poor results by 16% (OR 0.84, 95% CI [0.34-2.13]) and 58% (OR 0.42, 95% CI [0.10-1.73]), respectively, compared to the posterior approach ( Figure 6 and Figure 7). However, these differences were not statistically significant (p = 0.72 and p = 0.23, respectively).

Cosmetic outcome

**Figure 8.**
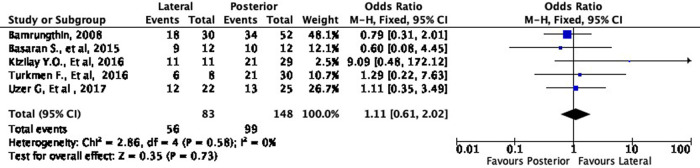
Comparison of cosmetic outcome in the excellent subgroup.

**Figure 9.**
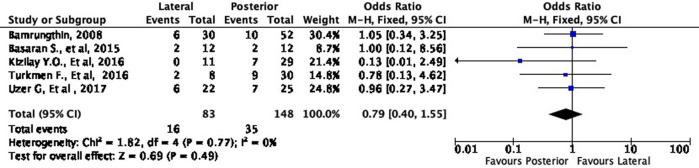
Comparison of functional outcome in the good subgroup.

**Figure 10.**
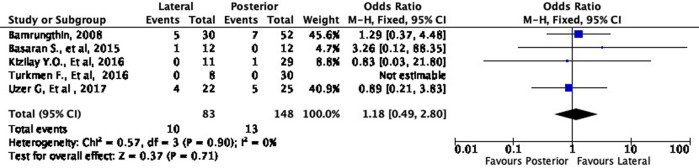
Comparison of functional outcome in the fair subgroup.

In terms of cosmetic results, both approaches showed mixed findings. The lateral approach was more likely to result in excellent (OR 1.11, 95% CI [0.61-2.02]) but less likely to result in good (OR 0.79, 95% CI [0.40-1.55]) cosmetic outcomes compared to the posterior approach ( Figure 8 and Figure 9). Interestingly, the lateral approach was also more likely to result in a fair (OR 1.18, 95% CI [0.49-2.80]) cosmetic outcome than the posterior approach ( Figure 10). However, none of these findings were statistically significant (p = 0.73, p = 0.49, and p = 0.71, respectively). In other words, the cosmetic outcome was relatively comparable amongst the two approaches. We did not perform a meta-analysis for the poor subgroup as only one study ^17^ reported the poor outcome; thus, it could not be compared.

## Discussion

Supracondylar humeral fracture (SHF) is a type of elbow injury in children that most often requires operative therapy than other injuries. 60 to 70% of all elbow injuries in children occur between five and seven years of age. ^1^ The SHF types that often require ORIF are those that fall into Gartland classification type 3 or 4. There is a shift from the fracture location in these types, making the configuration very unstable. Moreover, the unsuccessful reduction of minimally displaced fractures (i.e., Gartland type 2) also needs ORIF. ^7^

The high incidence of SHF makes the decision of which surgical approach to perform crucial. Surgical approaches to manage elbow injuries can be performed with the anterior, lateral, medial, or posterior approach. There is no clear evidence of which approach is superior based on the functional, cosmetic, and radiological outcomes. Some of the surgical approaches that are commonly performed are the lateral and posterior approaches. Our study found that the lateral approach gave superior results to the posterior approach in the excellent subgroup assessment using Flynn's criteria for functional and cosmetic outcomes. It was also superior in the good subgroup for functional outcome. However, these differences were not statistically significant. A lateral approach is an approach with the least exposure to the elbow's essential structures than other approaches. It also has fewer surgical wounds that could interfere with elbow joint range of motion. ^20^

This study also found that the lateral approach resulted in fewer poor outcomes than the posterior approach as evaluated using Flynn's criteria in the functional and cosmetic assessments. In other words, the lateral approach had an overall better result than the posterior approach, but this difference was not statistically significant. There is a considerable amount of damage to the triceps muscle in the posterior approach, which can interfere with the muscle's function postoperatively, causing as high as 6% muscle strength reduction compared to preoperative conditions. ^15^

The lateral surgical approach is quite popular because it has the least risk of damaging vital structures such as the ulnar nerve, brachial artery, and capsule ligament at the elbow compared to other surgical approaches. From the cosmetic point of view, the lateral approach’s surgical wound is preferred because it is less visible than the other approaches. Moreover, the lateral approach is a safe approach due to the good visual field of the elbow anatomy and adequate exposure to the radiocapitellar compartment. This approach is easily carried out through the internervous plane, which minimizes nervous injury so that the risk of iatrogenic nerve damage is minimal. Besides, the lateral approach has a better fracture perspective than other approaches. ^20^^–^^23^

In addition, the lateral approach is safer because less soft tissue is dissected, avoiding ulnar nerve damage. In cases requiring ORIF, the lateral approach is minimally invasive with minimal soft tissue dissection compared to the posterior approach. This is associated with the dissection or division of the triceps muscles, which often experiences more postoperative adhesions. ^23^ However, soft tissue swelling is frequently found in the lateral approach, especially when combined with the medial approach to obtain better surgical exposure. Still, there is no consensus on which approach is superior. ^24^ In addition, patients treated with a lateral approach tend to have fewer unstable fractures, complications, and re-operations. Previous research has shown that the lateral approach results are very satisfying, which shows that approximately 67-91.8% of them were successful. ^25^ This finding is similar to the study conducted by Sarrafan et al., who reported that 90.9% of 33 patients who underwent the lateral approach obtained excellent results. ^23^

Meanwhile, on the other hand, the posterior surgical approach is popular because it has a shorter operating time compared to other approaches. ^19^ However, this surgical approach is sometimes avoided by some surgeons because the triceps muscle is damaged in the process of reaching the fracture line. ^26^ Nevertheless, a study conducted by Chen et al. reported the posterior approach’s superiority compared to anterior and medial approaches in terms of surgery duration and blood loss during elbow surgeries. They found that the shortest surgery duration was the posterior approach (62.9 ± 7.4 minutes), which was shorter than the anterior and medial approaches (64 ± 7.6 and 73.7 ± 7.3 minutes, respectively). Besides, the posterior approach resulted in less blood loss compared to the anterior and medial approaches (135.8 ± 44.7, 147.1 ± 42.7, and 171.3 ± 34.6 ml, respectively). ^27^

In the present study, both lateral and posterior surgical approaches resulted in satisfactory results in more than 90% of the cases analyzed. Although the posterior approach has been associated with several complications such as decreased strength of triceps muscles, previous studies have shown that the functional and cosmetic results were comparable to medial and lateral approaches. Moreover, the posterior approach’s advantage such as a wider surgical field of view allows a trouble-free reduction process, resulting in shorter surgery time. Thus, the posterior surgical approach should always be considered whenever appropriate. ^28^

The limitation of the current study is the language restriction to only English-language articles; thus, we may have missed other eligible studies written in other languages. Another limitation is the low number of studies that were included in the analysis. Moreover, all of the included studies were level III studies. Thus, the present review’s evidence level may not be the highest, as we did not find any randomized controlled trials (RCTs). However, we believe that our search strategy was comprehensive and robust. Moreover, we conducted a thorough bias analysis based on the Cochrane recommendation. Thus, our results represent the current best evidence on this topic. Future studies should conduct high-quality original research, preferably RCT, to provide better evidence. Moreover, a study comprising a direct comparison of all existing approaches for SHF management is still needed.

## Conclusion

Both lateral and posterior surgical approaches resulted in satisfactory functional and cosmetic outcomes according to Flynn’s criteria. The two surgical approaches were comparable in terms of giving desirable functional and cosmetic outcomes for the management of SHF in children. However, the choice of surgical approach preference should be based on surgeons’ consideration in accordance with their experience and expertise.

## Reporting guidelines

Figshare: PRISMA checklist and flowchart for 'Lateral versus posterior surgical approach for the treatment of supracondylar humeral fractures in children: a systematic review and meta-analysis'. https://doi.org/10.6084/m9.figshare.14740545.v1. ^29^

## Data availability

All data underlying the results are available as part of the article and no additional source data are required.

### Extended data

Figshare: Appendix 1 search strategy. https://doi.org/10.6084/m9.figshare.14740584.v1. ^30^

Data are available under the terms of the Creative Commons Attribution 4.0 International license (CC-BY 4.0).
