# Supplementary material for: Lateral versus posterior surgical approach for the treatment of supracondylar humeral fractures in children: a systematic review and meta-analysis
Source: F1000Res. 2022 Jan 10;10:573. Originally published 2021 Jul 15. [Version 3] doi: 10.12688/f1000research.53599.3 (PMC8764559; doi:10.12688/f1000research.53599.3)
Supplement: Peer review history

# Peer review: Reviewer response for version 3

**DOI:** 10.5256/f1000research.117948.r119235

**Published:** 2022-01-17

**Authors:** Prateek Behera

**Affiliations:** 1 Department of Orthopaedics, All India Institute of Medical Sciences (AIIMS), Bhopal, Bhopal, Madhya Pradesh, India

The authors have explained their points well. I still consider that the authors should modify the following sentence of the discussion: "the posterior surgical approach should always be considered whenever appropriate" as "the posterior surgical approach could be preferred whenever appropriate". "always" is a strong word in scientific writing and should be used judiciously.

Are the rationale for, and objectives of, the Systematic Review clearly stated?

Partly

Is the statistical analysis and its interpretation appropriate?

Partly

Are sufficient details of the methods and analysis provided to allow replication by others?

Yes

Are the conclusions drawn adequately supported by the results presented in the review?

Partly

Reviewer Expertise:

Pediatric Orthopaedics, Deformity Correction and Complex Trauma.

I confirm that I have read this submission and believe that I have an appropriate level of expertise to confirm that it is of an acceptable scientific standard.

# Peer review: Reviewer response for version 2

**DOI:** 10.5256/f1000research.79450.r100535

**Published:** 2021-12-14

**Authors:** Prateek Behera

**Affiliations:** 1 Department of Orthopaedics, All India Institute of Medical Sciences (AIIMS), Bhopal, Bhopal, Madhya Pradesh, India

The authors have edited the text to make it more relevant now. However, a few concerns still remain.

1. They have mentioned the indications for CRPP as Gartland Type 3 and 4 fractures but as is well known, Gartland Type 4 fractures are diagnosed during surgery for Gartland Type 3 fractures when the surgeon finds out that the fracture is unstable in both flexion and extension. Thus, it is an intra-operative diagnosis. One does not diagnose Type 4 fractures on injury films. Considering that the authors have mentioned the argument that open reduction is used in situations where the C arm is unavailable, I believe using Type 4 along with Type 3 as an indication is inappropriate. The surgeon can never diagnose a Type 4 fracture in absence of an intra-operative C arm.
2. In the introduction, there is a spelling mistake when the authors have used the term 'medical' instead of 'medial'.
3. The authors have mentioned the disadvantage of the anterior approach as being more challenging but have not mentioned those of the other approaches. A reader deserves a balanced analysis and thus, they should mention the disadvantages of other approaches too. Injury to the capitellar blood supply is one such disadvantage for example.
4. The inference made in favor of the posterior approach in the discussion as "Thus, the posterior surgical approach should always be considered whenever appropriate" is inappropriate in my opinion as the results of the lateral and posterior approaches both based on this study are comparable.

Are the rationale for, and objectives of, the Systematic Review clearly stated?

Partly

Is the statistical analysis and its interpretation appropriate?

Partly

Are sufficient details of the methods and analysis provided to allow replication by others?

Yes

Are the conclusions drawn adequately supported by the results presented in the review?

Partly

Reviewer Expertise:

Pediatric Orthopaedics, Deformity Correction and Complex Trauma.

I confirm that I have read this submission and believe that I have an appropriate level of expertise to confirm that it is of an acceptable scientific standard, however I have significant reservations, as outlined above.

# Response

**Published:** 2021-12-18

**Authors:** komang irianto

**Affiliations:** Airlangga University, Indonesia

The authors would like to thank Dr Prateek Behera for the meticulous reviews. Our responses for the remaining concerns are:

 1. We agree with the reviewer’s comment that Gartland type 4 fractures are mostly diagnosed during surgery when instability is found (during reduction attempts/manipulation) in both flexion and extension in Gartland type 3 fractures. Nonetheless, as recent literature has shown encouraging results regarding predictive preoperative radiographic features of Gartland type 4 fractures ^1–3^, the authors suggest that the assumption of “not being able to diagnose Gartland type 4 fracture in the absence of an intra-operative C arm” should be reconsidered.

 Mitchell *et al*. identified five significant preoperative radiographic features associated with an increased likelihood of Gartland type 4 fractures. The most strongly associated feature was flexion angulation of the distal fragment (OR 17, 95% CI [4.9-59], p<0.001), followed by valgus angulation (OR 5.6, 95% CI [1.6-20], p=0.008), lateral translation of distal fragment in the coronal plane (OR 4.1, 95% CI [1.6-11], p=0.004), the presence of osseous apposition between proximal and distal fracture fragments (OR 4.0, 95% CI [1.8-9.0], p=0.001), and propagation of the fracture line toward proximal segment’s diaphysis (OR 9.2, 95% CI [1.6-53], p=0.01). ^1^

 Likewise, Barik *et al.* reported that a preoperative radiographic finding manifesting as valgus angulation of the distal fragment ≥17 ^o^ was a significant predictor of Gartland type 4 fractures (OR 20.22, p<0.001, sensitivity 88%, specificity 81%) ^2^. Meanwhile, Soldado *et al.* recommended that Gartland type 4 fractures should be suspected when the distal fragment is vertically aligned in lateral radiographs despite complete cortical disruption ^3^.

 In light of the justification above, the authors believe that Gartland type 4 is an appropriate indication along with type 3 in this study.

References:

1. Mitchell SL, Sullivan BT, Ho CA, Abzug JM, Raad M, Sponseller PD. Pediatric Gartland Type-IV Supracondylar Humeral Fractures Have Substantial Overlap with Flexion-Type Fractures. J Bone Joint Surg Am. 2019 Aug 7;101(15):1351–6. https://doi.org/10.2106/JBJS.18.01178
2. Barik S, Singh G, Maji S, Azam MQ, Singh V. Preoperative Prediction of Gartland IV Supracondylar Fractures of Humerus: Is it  Possible? Rev Bras Ortop. 2021 Apr;56(2):230–4. https://doi.org/10.1055/s-0040-1722578
3. Soldado F, Hodgson F, Barrera-Ochoa S, Diaz-Gallardo P, Garcia-Martinez MC, Ramirez-Carrasco TR, et al. Gartland Type-IV Supracondylar Humeral Fractures: Preoperative Radiographic Features  and a Hypothesis on Causation. Orthop Traumatol Surg Res. 2021 Sep;103049. https://doi.org/10.1016/j.otsr.2021.103049

2. We would like to thank the reviewer for his correction. We have amended the typing mistake in the newer version.

 3. The disadvantages of the four approaches are explained in several parts of the manuscript. In the Introduction part (Paragraph 3), the authors have mentioned the disadvantages of the medial approach (i.e., extensive soft tissue exposure/more scars) and anterior approach (i.e., neurovascular injury risks). However, we did not specify the neurovascular structure that could be injured in the previous version. We have amended the text by including median nerve and capitellar blood supply injury risks in the anterior approach.

 Meanwhile, the disadvantages of lateral and posterior approaches are stated in the Discussion part:

- Paragraph 5 of the Discussion part (highlighting the disadvantage of the lateral approach): *“…soft tissue swelling is frequently found in lateral approach.”*
- Paragraphs 6 and 7 of the Discussion part (highlighting the disadvantages of the posterior approach): *“…the triceps muscle is damaged in the process of reaching the fracture line” *and *“…the posterior approach has been associated with several complications such as decreased strength of triceps muscles.”*

4. The sentence in paragraph 7: *"Thus, the posterior surgical approach should always be considered whenever appropriate"* is an adjunct to the whole paragraph in discussing the posterior approach and not to conclude that the posterior approach is superior to the lateral approach.  In the Conclusion part, we have mentioned that since *"The two surgical approaches were comparable in terms of giving desirable functional and cosmetic outcomes for the management of SHF in children"*, we concluded that *"The choice of surgical approach preference should be based on surgeons' consideration in accordance with their experience and expertise."*

# Peer review: Reviewer response for version 1

**DOI:** 10.5256/f1000research.57005.r92398

**Published:** 2021-09-21

**Authors:** Prateek Behera

**Affiliations:** 1 Department of Orthopaedics, All India Institute of Medical Sciences (AIIMS), Bhopal, Bhopal, Madhya Pradesh, India

Based on the provided objectives of the study, the authors have attempted to find out the best approach for open reduction of the supracondylar humerus (SCHF) in children. While the authors have followed all the recommended steps for conducting a systematic review, the actual usefulness of this review for a practicing pediatric orthopedic surgeon dealing with SCHFs is a bit limited. The reasons for this observation are mentioned below:

1. The study looks at only two approaches for a common fracture - the lateral and posterior approaches - but has missed out on the anterior and medial ones. The indications for an open reduction include irreducible fractures and fractures with neuro-vascular involvement. Wingfield *et al*. ^1^ have described the details of how an open reduction becomes necessary in many cases and how an approach can be chosen. In their review, they have mentioned the advantages and disadvantages of different approaches in detail. Reitman *et al*. ^2^ studied their patients with SCHF and have mentioned that they prefer to approach through the ruptured periosteum so as not to injure the intact periosteum. If one follows this concept, then also the anterior approach might be the commonest one to be used. Thus, there is a selection bias in the study with the choice of approaches being only two. Ideally, the authors should have chosen studies on all the approaches and then performed the analysis. The result coming out of that comprehensive systematic review and meta-analysis would be the one that most pediatric Orthopaedic surgeons would like to see.
2. The stated objective of the study was to identify the best approach. Ideally, the best approach would be the one with the least difficulty in exposure, the best chances of providing a near anatomical reduction, the least number of complications, the best post-operative radiological outcome, and the best clinical and functional outcomes. Unfortunately, this study has tried to answer the research question using only one tool - the clinical and radiological outcome (using the system proposed by Flynn *et al*.). The authors should have included the complications, fixation failures, and other related issues in addition to the Flynn criteria.

In view of the two major points made above, in my opinion, the authors should be encouraged to expand the scope of the analysis and include all the approaches and focus on difficulties encountered, complications, reduction quality, loss of reduction, and clinical and functional outcomes. This would make the study more useful.

Are the rationale for, and objectives of, the Systematic Review clearly stated?

Partly

Is the statistical analysis and its interpretation appropriate?

Partly

Are sufficient details of the methods and analysis provided to allow replication by others?

Yes

Are the conclusions drawn adequately supported by the results presented in the review?

Partly

Reviewer Expertise:

Pediatric Orthopaedics, Deformity Correction and Complex Trauma.

I confirm that I have read this submission and believe that I have an appropriate level of expertise to state that I do not consider it to be of an acceptable scientific standard, for reasons outlined above.

## References

[1] : Open Reduction Techniques for Supracondylar Humerus Fractures in Children. *J Am Acad Orthop Surg* .2015;23(12) : 10.5435/JAAOS-D-15-00295 e72-80 10.5435/JAAOS-D-15-00295 26507292

[2] : Open reduction and internal fixation for supracondylar humerus fractures in children. *J Pediatr Orthop* .21(2) :157-61 11242241

# Response

**Published:** 2021-11-13

**Authors:** komang irianto

**Affiliations:** Airlangga University, Indonesia

**RESPONSE TO REVIEWERS**

 Dear Dr. Prateek Behera,

 The authors would like to thank you for reviewing our submission.

**Questions:**

1. The study looks at only two approaches for a common fracture - the lateral and posterior approaches - but has missed out on the anterior and medial ones. The indications for an open reduction include irreducible fractures and fractures with neuro-vascular involvement. Wingfield et al. have described the details of how an open reduction becomes necessary in many cases and how an approach can be chosen. In their review, they have mentioned the advantages and disadvantages of different approaches in detail.
2. Reitman *et al.* studied their patients with SCHF and have mentioned that they prefer to approach through the ruptured periosteum so as not to injure the intact periosteum. If one follows this concept, then also the anterior approach might be the commonest one to be used. Thus, there is a selection bias in the study with the choice of approaches being only two. Ideally, the authors should have chosen studies on all the approaches and then performed the analysis. The result coming out of that comprehensive systematic review and meta-analysis would be the one that most pediatric Orthopaedic surgeons would like to see.

**Answer:**

 As stated in the manuscript, we compared the results of lateral and posterior approaches, given that the two approaches are the most common approaches performed by surgeons in a limited setting where C-arm is unavailable. The gold standard management of supracondylar fracture, which is closed reduction followed by percutaneous pinning, could not be performed. The open surgery is planned based on the clinical pathway of supracondylar fracture. Anterior approach is mandated when vascular lesion is suspected. We have added this information to the manuscript.

**Question:**

1. The stated objective of the study was to identify the best approach. Ideally, the best approach would be the one with the least difficulty in exposure, the best chances of providing a near anatomical reduction, the least number of complications, the best post-operative radiological outcome, and the best clinical and functional outcomes. Unfortunately, this study has tried to answer the research question using only one tool - the clinical and radiological outcome (using the system proposed by Flynn et al.). The authors should have included the complications, fixation failures, and other related issues in addition to the Flynn criteria.

**Answer:**

 The main outcomes of supracondylar humeri management are function and cosmetic as established by Flynn *et al.*, where surgeons and patients could objectively evaluate the outcomes. The follow up time in months was reported by all included studies. The fixation failure, skin tract infection, and other related complications had been treated promptly and were considered a part of the functional and cosmetic outcomes.

# Peer review: Reviewer response for version 1

**DOI:** 10.5256/f1000research.57005.r89737

**Published:** 2021-08-19

**Authors:** Yudha Mathan Sakti

**Affiliations:** 1 Department of Orthopaedics and Traumatology, Dr. Sardjito General Hospital, Faculty of Medicine, Public Health, and Nursing, Gadjah Mada University, Yogyakarta, Indonesia

Congratulations on the paper, the controversies regarding the topic of comparing approaches for supracondylar fractures in children is clinically relevant.

 This study has an interesting background that evaluates the management of supracondylar humeral fracture (SHF) in children with the main concern of chosen approaches. Focus descriptions of comparing the lateral and posterior approaches for SHF with systematic review can be applied for decision making in the clinical setting; therefore, making this study a valuable contribution for managing SHF as the most common type of fracture in children.

 Specific description regarding the study may improve the narrative and enlighten better of the objective.

**Introduction:**

- The significant importance of good management for SHF is needed and well described in the introduction. The authors also describe the type of approaches and options to use and their limitation. Improvements are needed to describe the benefits and shortcomings in each approach to better explain the objective of this paper in comparing the lateral and posterior approaches.

**Methods:**

- The inclusion criteria used to select the studies to be enrolled in this paper is rigorous and well thought; however, the authors' reason to use Flynn’s criteria as a comparative measure between studies is needed.
- Description of whether each of the chosen papers directly used the criteria or the authors’ method of conclusion to generate the papers’ findings to be put in Flynn’s classification is still needed.

**Results:**

- Robust evaluation and selection are showed with the final 5 case-control studies included in this study. Description and bias evaluation is beneficial and creates a strong perspective to support the findings.
- From Table 4 (Patients’ outcome based on Flynn’s criteria), the authors conclude no significant difference on each functional and cosmetic findings between each paper in the qualitative analysis. This result is well depicted and described the objective of this study. Despite the findings, the authors’ method on how to conclude each paper in the qualitative evaluation is needed to provide a better description.
- Classifying each functional and cosmetic result from each paper and quantitatively compare them give a good insight into this literature review. These findings will give a beneficial perspective between the two approaches with methodical guidance led by this study.

**Discussion and Conclusion:**

- The study answered the objective with good methodical reasoning. Significant knowledge and rationale of each approach is well described. Advantageous and limitations for each approach is thoroughly explained with correlation to this study’s finding. Overall, this study will give valuable inputs and contributions to clinicians based on the conducted literature review.

Are the rationale for, and objectives of, the Systematic Review clearly stated?

Yes

Is the statistical analysis and its interpretation appropriate?

Yes

Are sufficient details of the methods and analysis provided to allow replication by others?

Yes

Are the conclusions drawn adequately supported by the results presented in the review?

Yes

Reviewer Expertise:

Orthopaedic and traumatology

I confirm that I have read this submission and believe that I have an appropriate level of expertise to confirm that it is of an acceptable scientific standard.
